# RobEns: Robust Ensemble Adversarial Machine Learning Framework for Securing IoT Traffic

**DOI:** 10.3390/s24082626

**Published:** 2024-04-19

**Authors:** Sarah Alkadi, Saad Al-Ahmadi, Mohamed Maher Ben Ismail

**Affiliations:** Department of Computer Science, College of Computer and Information Sciences, King Saud University, Riyadh 11362, Saudi Arabia; salahmadi@ksu.edu.sa (S.A.-A.); mbenismail@ksu.edu.sa (M.M.B.I.)

**Keywords:** adversarial machine learning, intrusion detection, Internet of Things, adversarial attacks, adversarial robustness

## Abstract

Recently, Machine Learning (ML)-based solutions have been widely adopted to tackle the wide range of security challenges that have affected the progress of the Internet of Things (IoT) in various domains. Despite the reported promising results, the ML-based Intrusion Detection System (IDS) proved to be vulnerable to adversarial examples, which pose an increasing threat. In fact, attackers employ Adversarial Machine Learning (AML) to cause severe performance degradation and thereby evade detection systems. This promoted the need for reliable defense strategies to handle performance and ensure secure networks. This work introduces RobEns, a robust ensemble framework that aims at: (i) exploiting state-of-the-art ML-based models alongside ensemble models for IDSs in the IoT network; (ii) investigating the impact of evasion AML attacks against the provided models within a black-box scenario; and (iii) evaluating the robustness of the considered models after deploying relevant defense methods. In particular, four typical AML attacks are considered to investigate six ML-based IDSs using three benchmarking datasets. Moreover, multi-class classification scenarios are designed to assess the performance of each attack type. The experiments indicated a drastic drop in detection accuracy for some attempts. To harden the IDS even further, two defense mechanisms were derived from both data-based and model-based methods. Specifically, these methods relied on feature squeezing as well as adversarial training defense strategies. They yielded promising results, enhanced robustness, and maintained standard accuracy in the presence or absence of adversaries. The obtained results proved the efficiency of the proposed framework in robustifying IDS performance within the IoT context. In particular, the accuracy reached 100% for black-box attack scenarios while preserving the accuracy in the absence of attacks as well.

## 1. Introduction

The internet has been growingly used by various computer applications and is continuously associated with several emerging technologies. This widespread use and exposure to diverse technologies widened the spectrum of potential attacks. In other words, even more luring targets are subjected to harmful attacks. Moreover, attacks within Internet of Things (IoT) environments that lack well-tailored security solutions proved to have substantial impacts. Intrusion Detection Systems (IDSs) have played an important role in ensuring IoT network security, subject to capacity and processing limitations [1,2]. In addition, Machine Learning (ML) techniques have been widely utilized to enhance the capabilities of several computer applications, including IDSs [3]. Typically, ML-based models are employed to infer hidden patterns and thereby perform proper predictions. However, such models are prone to performance degradation when Adversarial Machine Learning (AML) is brought into play [4]. In fact, AML proactively determines potential security threats and simulates realistic attacks using adversarial examples (AEs). Especially, several crafting methods of AEs are conveyed to affect the detection performance and cause model failure. Additionally, AML investigates promising defense mechanisms to overcome relevant issues such as standard accuracy sensitivity and AEs transferability to ensure robust performance [5].

Recently, several studies [6,7,8,9,10,11] were introduced to investigate the impact of AML techniques on ML-based IDSs. These works considered AML attack strategies, defense strategies, or both for the sake of robustness enhancement. In terms of attacks, gradient-based methods have been widely adopted because of their strength and the promising results they yield. Similarly, Particle Swarm Optimization (PSO)-based traffic mutation algorithms and GANs [2,3] have been coupled with AML attack strategies. With regards to defense, only a few generic defense techniques, such as adversarial training, have been introduced. 

Despite these efforts, AML has not been investigated thoroughly within the IoT context [5,12,13,14,15,16]. Very limited works investigated such issues and considered relevant domain constraints. This confirms the necessity to investigate the main elements of a robust ensemble adversarial machine learning framework in the IoT traffic context. Most of the related works investigated ML solutions for IoT-based IDS without consideration for potential vulnerabilities [17,18]. From an attack perspective, AML aims at shaping a more realistic attack setting by assuming the unknown learning process of the target model and the secure deployment of IDS. This is presumed due to the potential adversary behavior that is less likely to have access to either the training data or the model’s hyperparameters [1,19,20]. From a defense perspective, an ideal situation is achieved by enhancing the intrinsic robustness of ML-based IDS while maintaining the standard accuracy of model performance. This is obviously critical due to the increasing number of adversarial attacks, which cause even more challenges for the target model when they are unknown. Defense methods incorporate specific solutions to the model-based methods, while the least attention is given to the data-based ones [21]. It is also hard to identify how good the defense is since it is evaluated within specific contexts against specific types of attacks. Moreover, maintaining the accuracy of legitimate examples is quite challenging after robustifying the model [22]. This confirms the research gaps that consist of addressing AML threats using ensemble methods where both attack and defense perspectives are considered. In particular, the defense perspective can be elaborated to incorporate a combination of defensive modules that forms the “defense-in-depth” security concept. The adoption of such ensemble methods increases the attack complexity, in terms of time, knowledge, and computational resources, for the adversary [16]. 

Motivated by these observations, this triggers the need for: (i) questioning the robustness of ML-based IDS with respect to real IoT network traffic; and (ii) investigating potentially tailored solutions to AML strategies.

Accordingly, this research aims at answering the following research questions in order to reinforce the detection performance:

▪ What are the main elements of a robust ensemble AML framework that enhances ML-based IDS in an IoT environment?
▪ How would black-box attacks be employed in hardening ML-based IDS within the IoT context?
▪ How can dual defense methods be associated with an ensemble AML framework for ML-based IDS in an IoT environment?
▪ Can an ensemble AML framework designed for IoT traffic intrusion detection enhance performance without affecting the accuracy of the original examples?

The proposed framework is designed to tackle the IDS issues faced by black-box AML attacks in the IoT context. In a nutshell, the key contributions of this research can be summarized as follows:

▪ Design and implementation of a robust ensemble framework, RobEns, that integrates attack and defense strategies for enhancing the detection rate of multi-class IDS models within the IoT context.
▪ Comprehensive investigation of state-of-the-art IDS models, namely, Support Vector Machine (SVM), Logistic Regression (LR), Multilayer Perceptron (MLP), and Random Forest (RF) [4]. In addition, two novel ensemble learning models are considered to improve the proposed system’s performance further. The experiments conducted to validate and assess the proposed work rely on three relevant benchmarking datasets, namely UNSW-NB15 [23], ToN-IoT [24], and Edge-IIoT [25].
▪ Realistic deployment of four state-of-the-art AML black-box attack strategies for evaluating adversarial accuracy. In particular, black-box settings of the Fast Gradient Sign Method (FGSM) [26], Carlini and Wagner (C&W) [27], Zeroth-Order Optimization (ZOO) [28], and HopSkipJump [29] attacks are utilized in this research.
▪ A novel dual defense methodology where data-based and model-based defense techniques are jointly considered for strengthening IDS performance and proving substantial advantages of the proposed framework. Namely, Feature Squeezing, Adversarial Training, and Ensemble Learning Models are considered.

The rest of the article is organized as follows: Section 2 outlines the related works in terms of models’ selection, attack methods, and defense strategies used for IoT-based IDSs. Section 3 details the proposed framework, while the experimental setup, experimental results, and discussions are presented in Section 4. Finally, Section 5 points out the conclusion and future work.

## 2. Background and Related Work

This section outlines the background relevant to this research and reviews the state-of-the-art ML-based IDSs. In addition, typical adversarial attacks and defense methods adopted for IoT networks are explored. Moreover, AML frameworks in the literature are investigated to ensure the proper understanding of the proposed ensemble-based framework. Finally, the research gaps and challenges addressed by this study are identified for proper positioning. 

### 2.1. ML-Based Intrusion Detection Systems for IoT Networks

Machine learning-based IDS approaches represent promising deployments of security tools that defend against cyberattacks. Researchers have exploited such solutions and demonstrated their success in detecting attacks targeting IoT devices. However, special consideration should be given to IoT workflow requirements, such as keeping the processing load of devices to a minimum. Both conventional machine learning and deep learning techniques have been used to support IDS adaptation to IoT networks. However, deep learning methods raise even more challenges due to the constrained IoT storage and processing capacity. 

Consequently, conventional ML-based approaches have been favored for such applications due to their simplicity, stability, and robustness [2,3]. In particular, the research approaches of conventional ML for IDSs in the IoT can be grouped into tree-based, clustering-based, probabilistic-based, and non-probabilistic-based categories. Specifically, tree-based algorithms achieved competing results and thereby have been adopted in several studies. Gad et al. [30] evaluated various ML methods for both binary and multi-class classification scenarios using the ToN_IoT dataset. The selected models include Decision Tree (DT), Random Forest (RF), Classification and Regression Tree (CART), Extreme Gradient Boosting (XGBoost), K-Nearest Neighbor (KNN), Support Vector Machine (SVM), Logistic Regression (LR), and Naïve Bayes (NB). The obtained results proved the outperformance of tree-based algorithms, followed by K-Nearest Neighbor (KNN), which also yielded promising results. Additionally, Alsaedi et al. [31] investigated several ML models using the ToN_IoT [20] dataset. Particularly, probabilistic approaches such as Naïve Bayes (NB) and Latent Dirichlet Allocation (LDA) classification algorithms showed less detection efficiency compared to RF, SVM, LR, and CART. Moreover, ensemble learning, which combines two or more base learners in order to reinforce the aggregated decision, was also utilized for IDSs. This learning paradigm was considered in several research studies and yielded improved IDS robustness. Thaseen et al. [32] employed ensemble learning methods based on KNN, SVM, and LR and obtained better results compared to the involved baseline approaches. The ensemble model achieved an accuracy of around 99% using the BoT-IoT [33] and ToN_IoT [24] datasets. It is worth noting that tree-based algorithms and ensemble learning are well-known for designing reliable IDSs in several surveyed studies [12].

Several studies have relied on deep learning algorithms and architectures as a primary tool for IDSs in the IoT context. In fact, deep neural networks imitate human brain structure through the use of units/neurons and layers as the main elements of the network architecture. Examples of the earliest deep learning architectures used in such contexts include Multi-Layer Perceptron (MLP). The researchers in [4] utilized MLP over four different IoT-based datasets, including UNSW-NB15 [23], ToN-IoT [24], BoT-IoT [33], and Edge-IIoT [25]. MLP outperformed all the other conventional ML models studied and showed promising results. 

Other deep networks exhibit more complex architectures with more hidden layers, units, and network parameters. The researchers in [34] adopted different architectures, such as Recurrent Neural Networks (RNNs) and Convolutional Neural Networks (CNNs), to achieve competitive results in terms of detection performance. Particularly, one should note that the efficiency of ML techniques is typically affected by the quality of the data. Precisely, context-relevance, size, accuracy, integrity, and consistency are major criteria that reflect the data condition. In other words, pre-processing techniques are required to ensure ultimate performance and reliable detection for IDSs [4]. As such, the initial evaluations carried out in [2,4] were extended in this study to develop a framework with adversarial strategies to offer cost-effective means.

### 2.2. AML Attacks and Defense for IoT-Based IDSs

Despite the benefits of employing ML techniques, IDSs remain subjected to vulnerabilities that increase misclassification rates. This increases attackers chances to evade IDS and perform “successful” attacks. Such attack scenarios can be categorized into: (i) white-box attacks, where the adversary performs attacks on a known learning process of the target model; and (ii) black-box attacks, where the adversary performs attacks on an unknown learning process of the target model [1,2].

It is worth noting that the attacks conducted during the model training cause a major performance degradation due to their significant impact on the learning process. This can be represented by a white-box attack scenario, which intends to reveal the whole learning process. On the other hand, black-box attacks targeting the testing phase are less harmful since the hackers are less likely to reach the model’s training dataset or its hyper-parameters [1,2]. However, these models are designed with security controls and deployed in secure environments. This confirms the importance of investigating black-box attack scenarios in order to enhance the robustness of ML-based IDSs [12]. 

Different white-box attacks, such as Jacobian Based Saliency Map (JSMA) [2], Fast Gradient Sign Methods (FGSM) [26], and Carlini and Wagner [27], were investigated by several research works. One can notice that most AML attack methods were associated with pattern recognition and image classification tasks, while few efforts were devoted to the IDS application. Additionally, several research works measured the applicability of such attacks in other domains, and they proved their effectiveness in the intrusion domain as well [4,19]. 

Taheri et al. [5] applied a white-box attack scenario in the IoT context using popular attack algorithms, including FGSM [26], DeepFool, and Projected Gradient Descent (PGD) [2]. The attacks proved their effectiveness against visualization-based botnet detection systems with a high success rate. Clements et al. [35] also utilized a white-box scenario with a selection of attacks covering FGSM [26], JSMA [2], C&W [27], and the Elastic Net Method (ENM). Their experiment proved the effectiveness of these attacks, which continue to inhibit classification performance, with a success rate reaching 100%. 

Black-box attacks are more practical for enhancing IDS robustness compared to white-box attacks. The characteristics, behavior, and workflow of the target IDS system usually remain hidden from the adversary. This reflects the suitability of black-box attacks, where queries take place to exploit target IDS vulnerabilities. Examples of this attack strategy include transfer-based attacks that use substitute classifiers running white-box attacks such as FGSM [26], JSMA [2], and other popular types but within black-box settings [6,36]. Qiu et al. [36] adopted FGSM [26] and JSMA [2] in a black-box setting where a substitute model is also used. The obtained results revealed the transferability feature of the adversarial examples generated by the considered attacks. In fact, the adversarial examples successfully affected the target model’s performance with a high attack success rate of 100%. Moreover, there are other black-box attacks in score-based and decision-based approaches [19]. Examples of these attacks include Zeroth-Order Optimization (ZOO) [28] and HopSkipJump [29], respectively. 

From AML defense perspectives, several strategies were introduced to enhance the model’s robustness against attacks in both a reactive and proactive manner. Commonly employed strategies include feature squeezing, adversarial training, network distillation, adversarial detection, and ensemble classifiers. Compared to other defense methods, adversarial training is the most employed defense method for enhancing IoT-based IDSs [5,12,13,14,15,16]. On the other hand, limited efforts were made to investigate the other defense strategies in the IoT context. Some studies [6,15] suggest the use of the ensemble learning approach in IDS contexts generally. However, limited consideration has been given to such an approach in the IoT context [37]. Moreover, few works employ ensemble defense methods that primarily focus on modifying the model itself without consideration of the other defense approaches [4].

### 2.3. AML-Based Frameworks

For secure deployment of IoT-based IDSs, the design of a robust framework that tackles adversarial attacks has emerged as an essential need. Robustness in such a context can be defined as the miniaturization of the ML-based model performance against adversarial examples [21]. The robustness has been studied with respect to two main aspects, including either attacks or defenses separately. However, only a limited number of works addressed the combinations of these two aspects. 

Several works investigated the application of AML using deep learning-based IDSs. In particular, Taheri et al. [5] proposed an ensemble-based defense system against adversarial examples for IDSs. It mainly encloses three elements: (i) a detector, (ii) an attack engine, and (iii) a defense mechanism. Specifically, the detector is a deep learning-based model used for visualization-based botnet detection. The considered attack engine combines gradient-based adversarial attacks and GAN-based adversarial attacks. For the defense mechanism, adversarial training is employed to retrain the detector, which proves its efficiency. For the defense mechanism, adversarial training is employed to strengthen the system by giving focus to model-based defense. A similar approach was introduced by Fu et al. [12] and Ibitoye et al. [14], but with consideration for IoT-based IDSs. The former research employed adversarial training, while the latter one suggested the role of feature self-normalizing in enhancing the robustness of model performance. Adversarial training provides better results compared to feature normalization. One should note that a white-box attack scenario is adopted only to evaluate the robustness of a deep learning-based model. However, such works did not include black-box attack scenarios or dual-based defense approaches. 

On the other hand, other works explored the employment of conventional machine learning-based IDSs. Anthi et al. [13] employed several target models, including Bayesian Network (BN) [13], Support Vector Machine (SVM) [13], Decision Tree (DT) [13], and Random Forest (RF) [13], in a white-box attack scenario. They were evaluated against gradient-based Denial of Service (DoS) attacks using an IoT network dataset. Adversarial training is adopted here, which also improves the model’s robustness against adversarial attacks. Vitorino et al. [15] studied the robustness of a non-differentiable model against adversarial attacks for IoT-based IDS using a black-box scenario. The robustness of multiple tree-based algorithms was evaluated against evasion adversarial attacks. For such attacks, relevant-context adversarial examples were created using an adaptative perturbation pattern method (A2PM). Adversarial training was also performed to improve its robustness, which shows its effectiveness against the employed attacks.

### 2.4. Poisitioning the Paper

This research paper is intended to bridge research gaps related to machine learning, adversarial machine learning, network security, and IoT. Overall, most of the state-of-the-art works consider white-box attack scenarios with a single defense method, particularly adversarial training. This implies the lack of black-box attack scenarios in the context of IDSs and specifically within the IoT domain. It also indicates the need to investigate the concept of “defense-in-depth” and examine its effectiveness in IoT-based IDSs.

Accordingly, the framework proposed in this paper differs from existing works in three key ways. Firstly, black-box attack scenarios are employed to craft sufficiently good adversarial examples and explore their transferability phenomenon. Secondly, the defense perspective is addressed in a more advanced manner. Specifically, the proposed defense approach combines dual-based defense methods as well as embedded defense methods. Lastly, we expand the selection of the dual-based defense approach to accommodate both data-based and model-based methods.

Consequently, all these areas of interest are handled together to orchestrate a robust framework based on ensemble adversarial machine learning that incorporates attacks and defense mechanisms. Moreover, it fulfills the need for a tailored solution where domain constraints are considered and thereby provides enhanced IoT-based intrusion detection systems (IDSs).

## 3. A Proposed Robust Ensemble Adversarial Machine Learning Framework for Securing IoT Traffic 

### 3.1. Framework Overview

**Figure 1:**
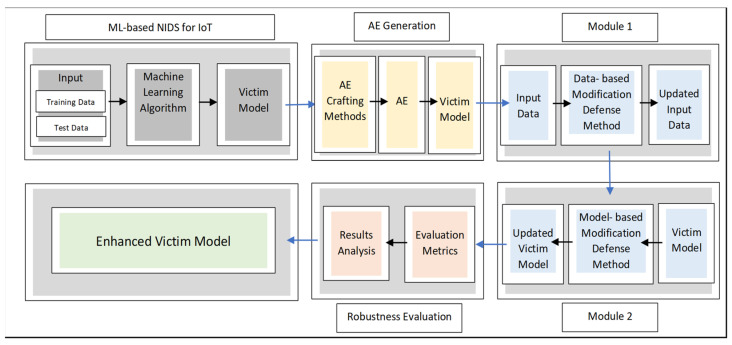
Proposed RobEns framework.

A security-by-design approach becomes essential during the development of intrusion detection systems. To assure trustworthy performance, such designs need to consider an attack-defense framework to ensure adversarially robust models. Moreover, the design specifications should be identified with consideration of IoT constraints in terms of time and space complexities [4]. Accordingly, as depicted in Figure 1, an ensemble approach is proposed and coupled with a defense-in-depth concept that considers (i) a single layer of attack methods and (ii) two layers of defensive techniques. The goal is to enhance the IDS’s robustness against potential attacks and avoid performance degradation in the context of IoT networks. 

**Figure 2:**
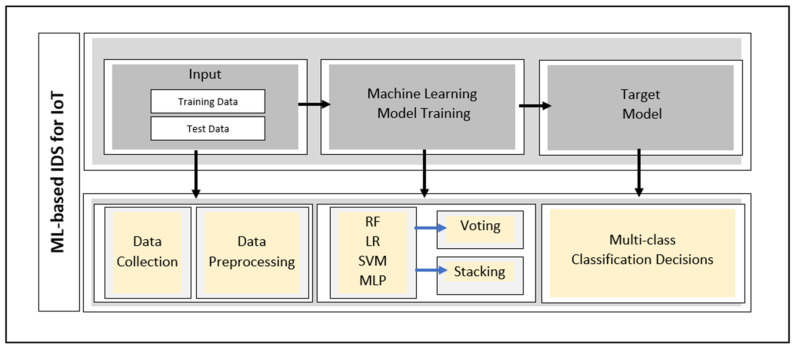
The ML-based IDS training process of the proposed framework.

Starting with the victim model’s selection, and given the IoT network resource constraints, conventional ML techniques are preferred. The robustness, stability, and simplicity of conventional techniques reflect their suitability compared to deep learning ones [4]. A multi-class classification scenario is adopted for detecting real-time attacks. The first phase of the framework is illustrated in Figure 2.

On the other hand, a black-box setting is employed to evaluate the robustness degree of the victim’s models towards the chosen attacks. In fact, the attack methods rely solely on three black-box types: (i) score-based methods, where a model’s class prediction is targeted; (ii) decision-based methods, where a model’s output label is targeted; and (iii) transfer-based methods, where a substitute model with white-box attacks is employed but in black-box settings. In particular, the black-box scenario is adopted due to the secure deployment of IDSs where strong security measures are used. This ensures that an adversary will not likely have access to neither the training set nor the model and its parameters [19,20]. Specifically, several state-of-the-art evasion attacks are utilized to measure the performance of victims’ models. 

Typical defense methods for IoT-based IDSs are presented as a single layer of defense using either data-based or model-based defense methods. However, combining the two approaches in order to further robustify the IDS system remains an open research gap. As such, the proposed RobEns encloses dual layers of defense stacked sequentially in order to increase attack complexity in terms of time and cost. These layers include data-based defense and model-based defense. Each of which represents an individual module within the ensemble adversarial machine learning framework. The first module consists of data-based modification defense methods, namely feature squeezing. Moreover, it focuses on input transformation through applying specific compression mechanisms to the feature space. On the other hand, the second module encloses a model-based modification defense method, namely adversarial training. This module augments the training phase of the model using adversarial examples generated by various attacks to reduce the attack’s effects on the model decision boundary. These two modules preserve the detection performance on benign examples in addition to adversarial examples, which tackle any potential bias issue and thereby achieve better generalization. 

### 3.2. Model Selection and Training for IoT-Based IDSs

In this work, two ensemble learning methods are proposed. Namely, stacking and voting alongside four base ML models selected from former studies. The four considered models proved to be effective in [4]. The selection of conventional machine learning methods implied their superiority in yielding competitive results while preserving less complex models and thereby satisfying potential resource constraints. They consume limited capacity for learning representation and require feature engineering steps to ensure solid decisions [3]. The following are the selected models, which include both conventional learning and basic deep learning methods:

- Logistic Regression (LR): A supervised probabilistic algorithm that performs classification using maximum likelihood. It relies on logistic sigmoids for both binary classification and multi-class classification tasks. It predicts the class of target value by assigning a threshold and output probabilities. A nonlinear transformation is applied to convert the absolute values into a range between zero and one [38].
- Support Vector Machine (SVM): A supervised non-probabilistic algorithm that performs both classification and regression tasks. It defines the dividing hyperplane where the input variable can be separated by the maximum margin. The hyperplane can be a linear or a nonlinear function of the input variable. This facilitates handling both binary and multi-class prediction using the kernel trick that includes linear, polynomial, and Radial Basis Functions (RBFs) [4,38].
- Random Forest (RF): A supervised ensemble tree-based algorithm that utilizes an army of tree-like structures for performing both classification and regression tasks. Each individual tree is trained on a randomly chosen subset from the training set. The quality of such trees is based on node split, where specific split criteria are employed, such as gini and entropy. The collective decisions of the tree army have very good accuracy in addition to fast and scalable performance on large datasets [4,38].
- Multilayer Perceptron (MLP): An early deep learning architecture that consists of an input layer, a hidden layer, and an output layer. This neural network contains connections between the nodes of each layer in a forward direction. The connection reflects a weighted summation of the previous layer that requires adjustment and fine-tuning using a backpropagation algorithm. The generalization of the model is achieved using multiple techniques such as weight decay, early stopping, dropout, and others [4,38].
- Ensemble Learning (EL): A collaborative learning method based on the wisdom of the crowd concept where collective decisions from multiple models are employed [12]. EL includes several main approaches, including “stacking” and “voting”. Stacking extends the bagging workflow by building a meta-model that combines, in an optimal manner, the final predictions of base models. Voting trains multiple base models in which initial predictions are determined independently by each model, and the final prediction is selected through averaging or majority voting [39]. In this study, stacking and voting are employed using *MLP*, *SVM*, and *LR* models that prove their efficiency individually in an IoT-based IDS context. This approach is assumed to construct stronger models that are harder to compromise compared to a traditional one [22].

Prior to the training phase, pre-processing mechanisms are applied to enhance the models’ performance. This includes discarding the least important features with no obvious contribution towards intrusion detection, such as origin and destination addresses. Additionally, data transformation is performed by employing one-hot encoding to turn the categorical features into numeric values. Then, data normalization is carried out using standard scalers, where feature scales are defined within the same range. For optimal configuration, the GridSearch [40] method is adopted for ensuring fine-tuned hyperparameters.

### 3.3. Adversarial Example (AE) Generation 

**Figure 3:**
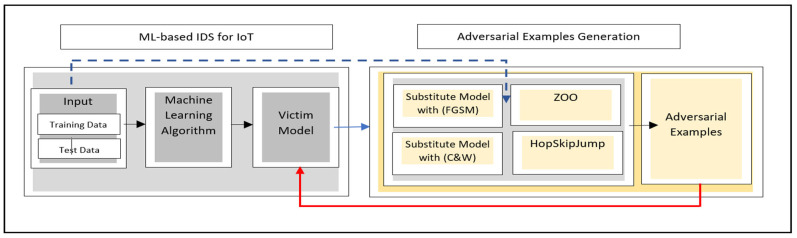
Generation of adversarial examples within the RobEns Framework.

The diversity of model types requires an attack scenario that can target both gradient and non-gradient models to ensure a reliable analysis and consolidated attack setting. As it can be seen in Figure 3, the proposed RobEns framework also takes into consideration the secure environment of IDSs and launches realistic attacks simultaneously in a black-box setting. 

Accordingly, four cutting-edge black-box methods are adopted to generate adversarial examples and analyze the detection performance. These attacks employ a non-targeted misclassification strategy where misclassifying to any false class is performed. The success rate of non-targeted attacks usually outperforms the targeted ones and is more common to be adopted. The attacks can be summarized as follows:

- Black-box Setting of the Fast Gradient Sign Method (FGSM) Attack: It is a transfer-based attack with a local substitute model trained using queried information from the target model. The method utilizes the sign of the gradient in calculating the cost function, thereby generating adversarial examples to mislead the target model. It is a typical white-box attack method that relies on a first-order projected gradient descent algorithm. The adversarial example is constructed using $l_{\infty }$-norm perturbation, taking into consideration the constraints on the maximal distortions. No iterative processes are performed, which yields its efficiency in terms of computational complexity [2,26]. Table 1 shows the attack parameters of the benchmark FGSM for all datasets.
- Black-box Setting of Carlini and Wagner (C&W) Attack: It can be introduced as a transfer-based attack with a local substitute model in which the output is used to mislead a target model. This attack is considered one of the strongest attacks where adversarial examples are constructed using the $l_{2}$-norm of Euclidean distance. The norm is applied for quantifying the difference between the original and adversarial examples, in which large distortions are penalized. The attack employs the Adam optimizer, starting points, and a tanh-nonlinearity function when performing an iterative targeted gradient attack. The attack has shown competitive performance in bypassing 10 detection methods intended to detect adversarial examples [19,27]. Table 2 shows the attack parameters of the benchmarks C&W for all datasets.
- Zeroth-Order Optimization (ZOO) Attack: It can be defined as a score-based attack with an assumption of access to the prediction confidence only and a performance as effective as the Carlini and Wagner white-box attack. This attack does not use the gradient nor the smoothness of the target model’s output. The method performs an attack by minimizing the distance between the decision boundary and benign examples. A zeroth-order optimization algorithm is used with a randomized gradient-free method to minimize such distance and formulate the attack [28,41,42]. Table 3 shows the attack parameters of the benchmark ZOO for all datasets.
- HopSkipJump Attack: It is a decision-based attack that relies on a novel estimation of gradient direction to generate adversarial examples given a defined perturbation range. The attack involves geometric progression and binary search for stepsize search and boundary search, respectively. The approach is hyperparameter-free for both targeted and untargeted attacks. Moreover, more complex settings, such as non-differentiable models or discrete input transformations, can be handled by this attack approach. Furthermore, it employs query-efficient algorithms that yield competitive performance for boundary attacks and strong defense mechanisms [22,29]. The parameters of the benchmark HopSkipJump attack considered for all datasets are reported in Table 4.

It is worth noting that $l_{\infty }$ distance is chosen, which is more suitable for evaluating defenses such as adversarial training [29].

### 3.4. Defenses against Adversarial Attacks

Defense methods for robustifying IDS models can be grouped into two main categories: data-based and model-based modifications. These methods are typically employed to ensure effective countermeasures for adversarial attacks. In this work, a proactive association of these two methods is employed to form an ensemble-based defense technique. Such a technique represents a “defense-in-depth” concept since a combination of defense methods is employed. For the proposed framework, feature squeezing and adversarial training methods are used in designing such modules, which are described as follows:

A. Module 1: Feature Squeezing-based Defense

In this module, feature squeezing is adopted as a data-based modification defense mechanism against adversarial example attacks. Specifically, it is used for reducing the feature space and thereby limiting the search space of potential adversaries’ perturbations. In fact, the feature space is highly dimensional, which causes vulnerabilities for ML-based solutions and eases the crafting of adversarial examples. The compression of the input features is followed by a comparison of the model’s predictions obtained using the original inputs and the compressed ones. One should mention that the input is considered adversarial if the difference between the two predictions’ results is considerable. Additionally, different compression methods such as bit depth compression, median smoothing, and non-local means can be utilized in this module [43,44].

B. Module 2: Adversarial Training-based Defense

In this module, adversarial training is employed as a model-based modification defense mechanism. It is used to strengthen the target model for efficient detection of adversarial examples. In particular, it trains the target model over a combined dataset that includes both original and adversarial examples. The adversarial examples are generated using different attack approaches and victim models, reflecting their unique transferability characteristics. This enhances the robustness of the trained target model to defend against possible AML attacks. Moreover, this contributes to avoiding overfitting and generalizing well over unseen inputs [2,42]. It is worth noting that the model-based modification IDS models have shown the most successful enhancement in terms of detection performance [2].

## 4. Experiments

This section starts by presenting the benchmarking IoT-based IDS datasets used for this study alongside the evaluation metrics to measure the detection performance. Then, the adopted classification scenarios are explained and analyzed among all the selected models. Moreover, the results achieved by applying black-box attacks to the proposed ML-based IDS models in multi-class classification scenarios are reported and discussed. Finally, a robustness analysis is conducted by investigating the performance of the proposed adversarial defense methods intended to enhance IDS models’ robustness.

### 4.1. Datasets and Evaluation Metrics

Classification scenarios for network intrusion detection generally, and IoT networks specifically, are categorized into binary and multi-class classification. Binary classification tackles the detection task by mapping traffic flow into either benign or malicious labels. On the other hand, multi-class classification distinguishes between several attack types based on their belonging category. The latter scenario exhibits a larger attacking spectrum, which makes the problem even more acute compared to binary classification tasks. As such, the multi-class classification scenario is considered in this research, where each model identifies each attack category and differentiates between the multiple classes. It is deployed using three IoT-based benchmarking datasets. Namely, UNSW-NB15 [23], ToN-IoT [24], and Edge-IIoT [25] datasets were considered for this research experiment.

UNSW-NB15 Dataset: This dataset was released in 2015 by the Cyber Range Lab of the Australian Centre for Cyber Security (ACCS) for the IDS context in general. Different tools are used to create network traffic and extract relevant features, including Perfect Storm [23], Argus [23], and Bro-IDS [23]. It contains hybrid network traces representing benign and malicious activities. Nine attack categories are identified, including analysis, backdoors, DoS, exploits, fuzzes, generic, reconnaissance, shellcode, and worms [23].

ToN-IoT Dataset: This dataset was released in 2019 by ACCS with a heterogeneous collection of relevant IoT-based traffic. Several resources were used in the creation of this dataset, including blur, cloud layers, edges, physical systems, and virtual machines. Attack categories span over different types, including: Several attack categories are defined, including backdoors, cross-site scripting (XSS), DoS, distributed DoS (DDoS), injection, man-in-the-middle (MITM), password cracking, ransomware, and scanning. It encompasses multiple subsets extracted from different operating system environments, such as Linux and Windows, along with traffic from networks and telemetry data from IoT services [24].

Edge-IIoT Dataset: This dataset was newly released for the purpose of analyzing heterogeneous data sources from both IoT and industrial IoT (IIoT). Different types of low-cost IoT digital sensors are employed to generate real traffic, including flame sensors, heart rate sensors, pH sensors, soil moisture sensors, ultrasonic sensors, temperature and humidity sensors, and water level detection sensors. An employment of 14 attack types is grouped into 5 main categories: DoS and DDoS attacks, information gathering, injection, man-in-the-middle (MITM) attacks, and malware attacks [25].

Accordingly, Table 5 summarizes the main characteristics of all the aforementioned datasets.

In terms of the characteristics of datasets, they incorporate context-related IoT traffic from both real and simulated network flows, including a mix of benign and malicious traffic. Moreover, different types of network attacks, such as Denial of Service (DoS), Distributed Denial of Service (DDoS), Brute Force, Botnet, Backdoor, and Injection attacks, are enclosed in these datasets. In particular, network traffic features are utilized to identify attacks and thereby enhance detection performance. The features are mainly grouped into four classes: (i) Basic features, which indicate traffic state, used protocols, service type, source-to-destination times and packet count, and destination-to-source times and packet count. (ii) Time-related features, which define traffic inter-arrival time, active-idle time, timestamp, source jitter, and destination jitter. (iii) Flow-related features that identify flow characteristics such as flow length, number of bytes, number of packets, bulk rate, downlink, and uplink ratio. (iv) Flag-related features that represent flag setting and counts in both forward and backward directions, such as SYN, URG, ACK, and FIN. (v) Connection-related features, which represent several connection settings, counts, and protocols, such as the Get and Post method in the http protocol, the ftp session login status, the number of bytes, and packets sent in both forward and backward directions.

In terms of evaluation, adding a degree of robustness is an important pillar in enhancing the detection of ML-based solutions for IDS. Robustness in the IDS context can be defined as the ability of machine learning solutions to decrease their susceptibility to adversarial examples [21]. Attacks and defenses are frequently associated with evaluating the robustness of machine learning solutions. These two aspects are employed together, representing a game scenario for augmenting robustness. This scenario involves measuring robustness based on defense methods effectiveness against attacks as well as the ability of attacks to crack such methods. This can preserve continuous learning and constant improvement [45,46].

In the literature, several performance measures are used in the analysis of the model’s robustness in both regular and adversarial holdout sets. With reference to adversarial examples, detection accuracy and attack success rate represent key elements in evaluating the performance from both attack and defense perspectives. In terms of accuracy, consideration is given in this study to the accuracy measure due to its reliability in relevant analyses. The accuracy identifies the correctly classified sample proportion of both benign and malicious types to reflect the detection performance of a specific model. It is derived indirectly from a confusion matrix that reports True Positive (*TP*), True Negative (*TN*), False Positive (*FP*) and False Negative (*FN*) values. The accuracy can be expressed as follows [47,48]:

(1) $$Accuracy\;(Standard,Adversarail,\;Robust)=\frac{TP+TN}{TP+FN+TN+FP}\times 100\;$$

where *TP* and *TN* reflect the successfully classified benign and malicious inputs, respectively, while the latter two values, *FP* and *FN*, indicate misclassified benign and malicious inputs. Moreover, accuracy is employed in measuring the performance of three components of the proposed framework, including the original classification, the classification under attacks, and the classification after applying defense methods. Thus, the metric measures the performance of the original holdout set to be further compared with performance of adversarial and robustified ones. There are three sub accuracy metrics for such a reason: standard accuracy (the model’s accuracy when there is no adversary), adversarial accuracy (the model’s accuracy when there is an adversary without defense), and robust accuracy (the model’s accuracy against an adversary with defense) [47,48].

Another measurement that is used to measure the level of compromising the target model integrity by attacks is the Attack Success Rate (ASR). It represents the total number of perturbed dataset inputs in which the adversarial examples cause misclassification by the target model [49]. This involves classifying the adversarial examples in their target class by the target model [50].

A higher attack success rate corresponds to lower adversarial accuracy [49]. It can be given by the following formula:

(2) $$Attack\;Success\;Rate\;\left(ASR\right)=1-Adevrsarail\;Accuracy$$

### 4.2. Classification Performance

In preparation for the classification task, all datasets were divided into 70% training and 30% testing sets in order to ensure better model generalization.

**Figure 4:**
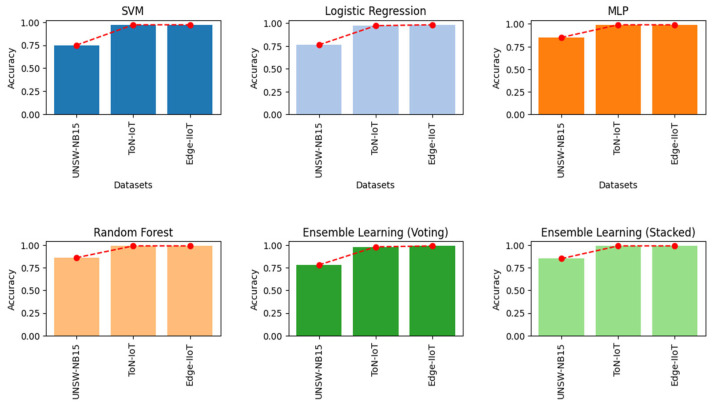
Performance of multi-class classification achieved by the considered models.

All of the aforementioned models were trained and evaluated using the three benchmark datasets. The obtained accuracy values are shown in Figure 4.

As can be seen, the reported results show a variation in performance among the different datasets, specifically UNWS-NB15 [23]. The models’ performance ranged from 74% to 77% for SVM, LR, and voting ensemble learning. The MLP and RF models exhibited better performance, ranging between 84% and 85%. The stacked ensemble learning model yielded the best performance with 86% accuracy. For ToN-IoT [24] and Edge-IIoT [25], the considered models showed better performance, with accuracy ranging from 97% to 99%. In particular, the accuracy achieved by the stacked model and ToN-IoT [20] reached 99%, while the lowest accuracy of 97% was recorded for LR. With regards to Edge-IIoT [25], RF yielded the highest accuracy of 99%, followed by the stacked model and MLP. The remaining classifiers yielded accuracy ranging from 97% to 98%. One should note that the lowest accuracy was obtained using the SVM model. All the results are summarized in Table 6.

### 4.3. Adversarial Attacks Performance against IDS Models

The four adopted attacks are applied in the context of multi-class classification scenarios. The scenario considered involves difficulty in crafting adversarial examples due to the large number of targeted classes. These attacks are initially employed for image modality, which is different from the tabular data typically used in the IDS domain. Thus, there is a need for normalization methods where a defined range for network features is used, resembling the image pixel value range. All the attacks obtain different performance in terms of adversarial accuracy and attack success rates, with either minor or major effects.

A. Adversarial Results

Table 7 shows the adversarial accuracy obtained using the selected models along with the UNSW-NB15 dataset [23]. For each attack, the detection performance exhibits a considerable variance for the different classifiers. In particular, a significant degradation is recorded for the HopSkipJump attack, followed by a substitute model coupled with the FGSM attack. The rest of the attacks yielded similar performance with moderate effects. On the other hand, the RF model achieved the highest adversarial accuracy. For this dataset, the results achieved by the ensemble learning models exhibit non-competing performance. In other words, ensemble learning itself was not robust enough.

Table 8 reports the models’ performance achieved under the four attacks using the ToN-IoT dataset [24]. As can be seen, the extreme deprivation is attained under the HopSkipJump attack with an adversarial accuracy ranging from 0% to 2%. On the other hand, the ZOO attack is the weakest attack in this experiment. As can be seen, minor accuracy changes are observed. One should also note that RF was able to resist better with an adversarial accuracy between 65% and 99%. Regardless of the HopSkipJump attack, the stacked model yielded the second-best adversarial accuracy, followed by LR. The rest of the models achieved similar performances, coupled with different levels of recorded effects.

Table 9 also outlines the adversarial accuracy obtained using the Edge-IIoT dataset [25]. As one can see, HopSkipJump keeps achieving the highest performance degradation, while ZOO yields the weakest attack. In particular, the attacks associated with substitute models, including FGSM and C&W, caused notable effects on the investigated models. However, RF outperformed the other models in terms of robustness, in which adversarial accuracy was decreased by only 20%. To sum up, different levels of degradation were recorded for all models’ adversarial accuracy under the considered attacks, except for the ZOO attack, which yielded a slight decrease. Moreover, there was a notable effect of substitute models’ attacks, which confirmed the effectiveness of adversarial examples transferability in degrading the performance of other target models. On the other hand, models such as RF, MLP, and stacked models exhibit more robust performance and higher adversarial accuracy.

Tree-based algorithms and ensemble learning are well-known for designing reliable IDSs in several surveyed studies [12]. However, RF shows better performance compared to ensemble learning, which indicates a potential investigation of employing RF as a base learner within ensemble learning methods [15,51]. It is worth noting that employing tree-based algorithms has obtained competitive performance in a wide range of applications due to several factors, including simplicity, interpretability, and efficiency [42]. This is inferred from the use of rule sets that are easy to interpret, analyze, and integrate into real-time technologies [52].

B. Attack Success Rate (ASR) Analysis

**Figure 5:**
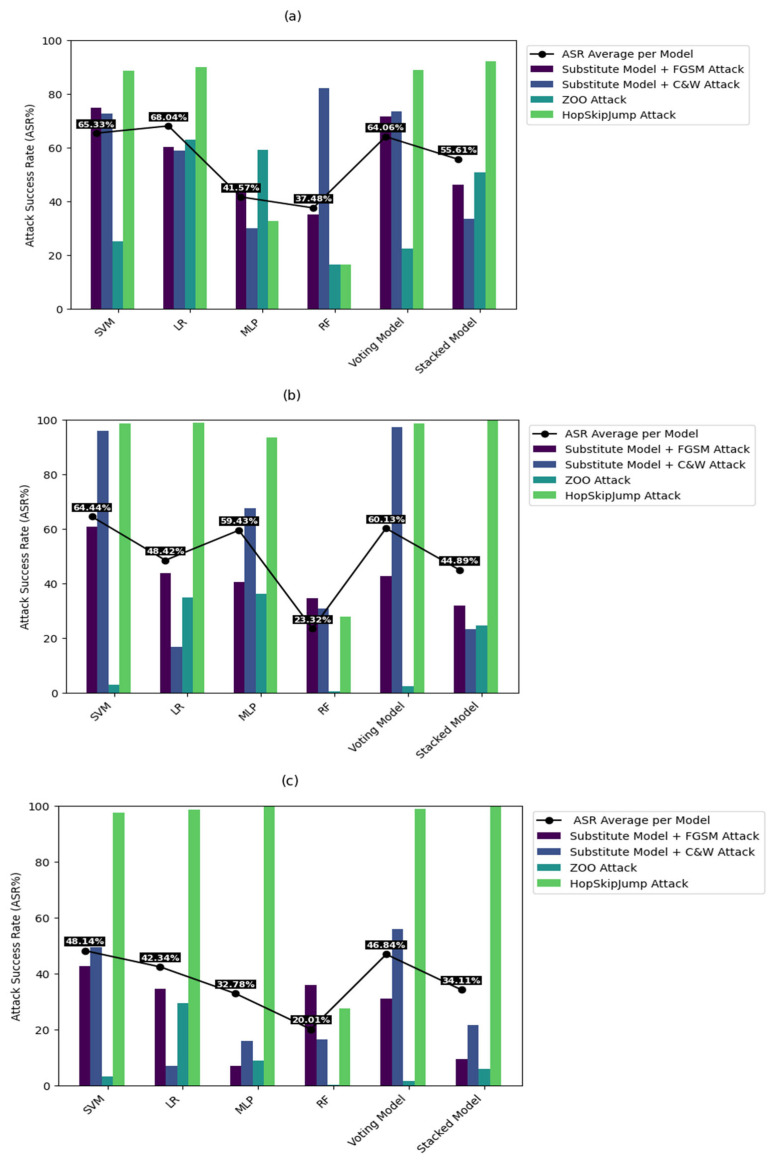
ASR rates obtained using the (**a**) UNSW-NB15 [23], (**b**) ToN-IoT [24], and (**c**) Edge-IIoT [25] datasets.

As shown in Figure 5, the substitute model increased the ASR considerably using all datasets when associated with the FGSM attack [26]. It yielded the third-best ASR out of the four studied attacks. This is expected due to the simple attacking method of FGSM [26] with one-single step adversarial sample generation. However, a substitute model coupled with a C&W attack [27] yielded the second-best ASR. This can be attributed to the complexity of such attacks. For the other black-box attacks, the ZOO attack [28] yielded the lowest performance of ASR. Moreover, for the three datasets, the proposed IDS models exhibit considerable performance degradation. Notably, the HopSkipJump attack [29] achieved the first-best ASR, reaching 100% successful exploitation. Accordingly, the HopSkipJump attack [29], followed by substitute models’ attacks, proved to be highly efficient in generating adversarial samples. As such, this can be considered a serious threat that requires the corrective action of different defense strategies. The performance of each attack against the different IDS models, including the average ASR, is jointly presented in Figure 5. Shedding the light on IDS models, they achieved similar performance with fluctuating attack success rates, except for the HopSkipJump attack [29], which attained a competitive ASR level. Obviously, the robustness and vulnerability of models vary based on the deployed algorithm, attack, and dataset types.

The xxRF model yielded low attack success rates ranging between 17% and 28%. On the other hand, SVM, MLP, and LR achieved fluctuating performances in terms of ASR. Additionally, the voting ensemble model achieved similar performance compared to the considered base learners, including SVM, MLP, and LR. Specifically, the ASR obtained using the voting model reflects fair results, with averages of 64%, 60%, and 46% for UNSW-NB15 [23], ToN-IoT [24], and Edge-IIoT [25], respectively. On the other hand, the stacked ensemble learning model gives lower attack success rates in comparison with the voting ensemble learning model. This reflects the better resilience of the model stacking method in such attack scenarios, with only a limited effect on detection accuracy. Stacking involves producing high-level learners based on the strengths of several levels of base learners with high generalized performance. However, the voting ensemble model generates the final decision based on averaging the predictions of the base learners. The weighted voting process is more prone to learning biases [53].

Accordingly, one can claim that the obtained results proved that RF, MLP, and stacked ensemble models achieved the most robust performance against three black-box attacks with the lowest ASR rates. It is worth noting that the ensemble model by itself represents a model-based modification defense mechanism. However, HopSkipJump showed the extreme weakness of the ensemble method, and further investigation of better algorithm selection is needed [42,54]. Obviously, the robustness and vulnerability of models vary based on the deployed algorithm, attack, and dataset types.

A venue for improvement in such a context includes the optimal selection of the base model and relevant hyperparameters to enhance the performance of the final ensemble learning model [55]. This is important to ensure that ensemble learning models are effective in hardening the IDS performance and complicating any potential attack attempt.

### 4.4. Adversarial Defense Performance for Robustifying IDS Models

In this experiment scenario, two-layer defense modules are employed for robustifying IDS against the four considered attacks. In particular, the two-layer approach includes feature squeezing and adversarial training as data-based modification and model-based modification defense methods, respectively. This approach starts with analyzing individual components performance as well as their combination as ensemble defense modules. One should recall that feature squeezing is used to compress the input features and compare them with the original inputs, mimicking the hash mechanism. Bit-depth compression is employed as a compression method and shows improvement in terms of robust accuracy. The conducted experiments showed the properness of this approach for IDSs. For the experiments, the parameters of the feature-squeezing defense method shown in Table 10 were adopted.

In terms of performance results, the method resulted in a notable improvement in robust accuracy, ranging between 5% and 80%. It is worth noting that feature squeezing is more effective in robustifying models against score-based and decision-based black-box attacks (i.e., ZOO and HopSkipJump) compared to transfer-based attacks with substitute models. However, considering feature squeezing as a standalone solution, it proved to be slightly effective when associated with transfer-based attacks. Moreover, the performance of the ensemble learning models remained unchanged in most cases or yielded a slight increase ranging between 1% and 2%. This indicates the lowest effectiveness of this defense method when ensemble learning models are deployed.

With regards to adversarial training, it is also employed for enhancing models’ robustness using the adversarial examples generated by the four studied attacks. The substitute models are used to approximate the decision boundaries of the target’s models due to the indirect utilization of powerful white-box attacks. The synthetic inputs generated by FGSM [26] and C&W [27] attacks were employed for adversarial training of the target models. Moreover, ZOO [28] and HopSkipJump [29] attacks were deployed directly to the target models, and their resulting examples were also employed for adversarial training. The key parameters of the adversarial training defense method used for all datasets, including classifier type, attack type, and number of training epochs, are listed in Table 11.

For performance evaluation, this method significantly improved the robust accuracy of the target models throughout the entire attack and, in some cases, exceeded its standard accuracy. However, the obvious increment in robust accuracy over the standard accuracy reflects the label leakage issue. This issue is associated with adversarial training defense methods when the accuracy of adversarial examples is higher than the accuracy of original examples [56]. This happened due to using the true label as input, especially while performing a non-iterative attack. In addition, slight drops in standard accuracy are noticed in some cases when applying adversarial training.

For ensemble learning, revisiting adversarial accuracy indicates the importance of reassessing the method selection for the base learner of ensemble models. The performance of the ensemble learning models varies depending on the selected base learners. In our experiment, the performance of the two proposed ensemble learning models that rely on LR, SVM, and MLP learners was below expectations. In particular, the voting ensemble model did not enhance adversarial accuracy, and its performance exceeded slightly in adversarial accuracy compared to the base learners. On the other hand, the stacked ensemble model outperformed the voting technique and yielded one of the top three best accuracies.

**Figure 6:**
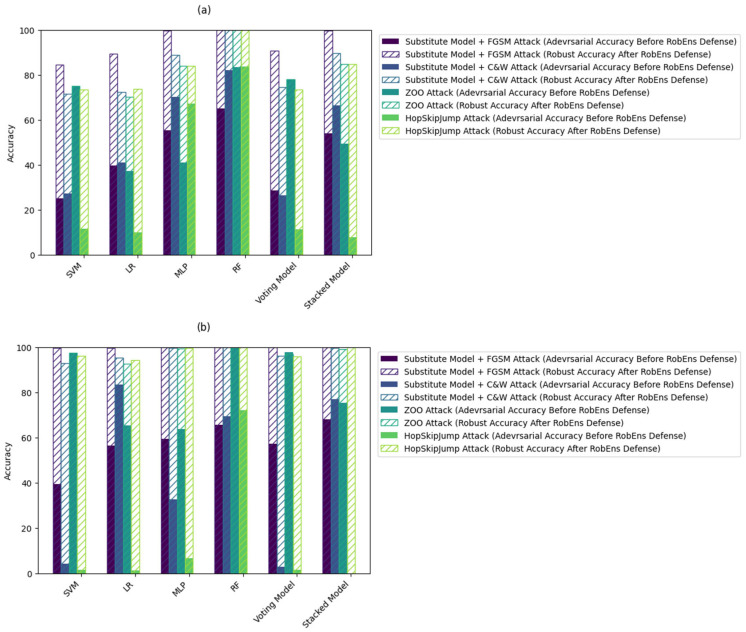

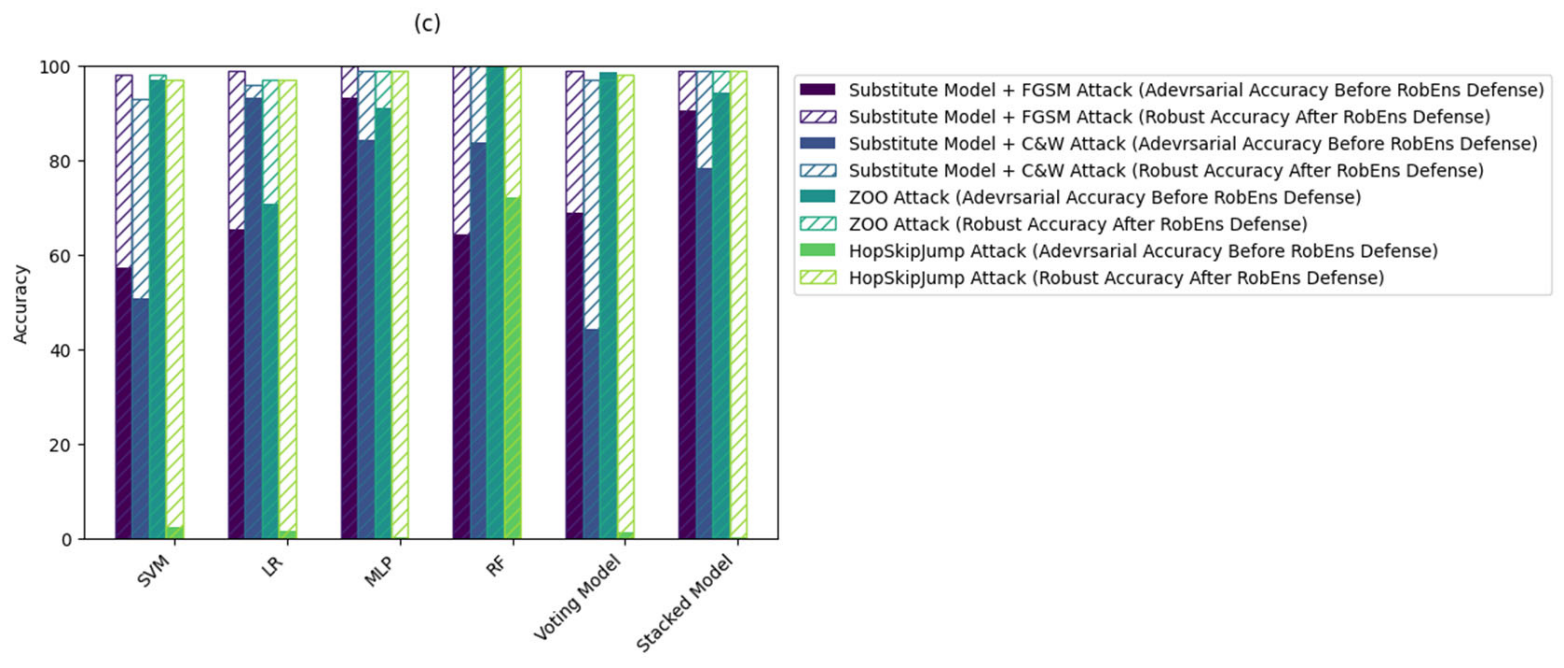
Accuracy obtained using different models before and after considering the RobEns, including the two-layer defense approach: (**a**) UNSW-NB15 [23], (**b**) ToN-IoT [24], and (**c**) Edge-IIoT [25].

For the proposed RobEns framework, combining the two defense methods, including feature squeezing and adversarial training, reveals a significant improvement in terms of robust accuracy while maintaining a better level of standard accuracy compared to the single defense method. On average, the robust accuracy increased between 70% and 100% for all the datasets and considered models. Figure 6 shows the robust accuracy for each model after adopting the RobEns defense framework (hatched bars in the upper part of the plot) and the adversarial accuracy before considering RobEns (bars in the lower part of the plot).

In terms of standard accuracy, Table 12 reports the standard accuracy recorded with and without adopting the proposed defense mechanisms. The results confirm the effectiveness of the proposed method in maintaining standard accuracy in the vast majority of experiment results. Frameworks ensure providing more secure frameworks to mitigate potential security vulnerabilities targeting IoT networks.

Overall, the two-layer defense methods implemented using the RobEns framework enhanced the resilience against attacks, improved the robust accuracy of the models, decreased adversarial accuracy, and mostly maintained the standard accuracy of the classification tasks. This can be inferred from Table 13, where a comparison between the performance of related works in Section 2.3 and the proposed method is presented.

## 5. Conclusions

In this research, a robust ML-based IDS framework is proposed, named RobEns, for defending against AML attacks in the context of IoT. Specifically, the framework incorporates both attack and defense perspectives. The latter one relies on two-layer defense modules, representing an ensemble approach intended to ensure high defense capability. Moreover, the proposed two-layer defense approach combines data-based and model-based defense methods, including feature squeezing and adversarial training, aiming at designing multi-based defense strategies. The proposed framework is designed by taking into consideration IoT limitations in terms of capacity and capability. Accordingly, the encompassed techniques exhibit simplicity, scalability, and manageability. Moreover, four state-of-the-art machine learning models—SVM, LR, MLP, and RF—were investigated, along with two ensemble learning models. The framework models were evaluated using four cutting-edge black-box attack methods. Namely, transfer-based attacks using FGSM and C&W, ZOO, and HopSkipJump were considered in addition to the three benchmarking datasets in the conducted experiments. The obtained results revealed the potential compromising of ML-based IDSs by adversarial attacks and the effectiveness of defense methods in ensuring the intended robustness. The robust accuracy of target models improved substantially by 30% to 100% using the proposed two-layer defense methods for the considered black-box attacks. Moreover, robustifying IDS models did not affect the standard accuracy, which was maintained at a level similar to the one achieved using legitimate examples. Additionally, slight decrements of nearly 7% were recorded in very few cases.

In the future, further investigation of advanced ensemble learning methods can be conducted. This represents a promising research direction to improve the proposed framework. Moreover, more efforts shall be devoted to diversifying base learners in order to enhance IDS robustness while reducing the computational overhead. This can incorporate the employment of federated learning to provide a more secure context and investigate its effectiveness within the AML domain. Some recent studies reveal promising results in enhancing the detection of IoT-based IDS through collaborative modeling. This involves considering the limitations of IoT resources by not requiring direct data sharing [57,58].

## Figures and Tables

**Table 1:** Summary of the considered FGSM configuration.

Parameter	Value
Norm ($l_{p}$)	$l_{\infty }$
Epsilon (ϵ)	0.1

**Table 2:** Summary of the considered C&W configuration.

Parameter	Value
Norm ($l_{p}$)	$l_{2}$
Confidence (κ)	0.0
Max iterations	1000
Binary Search Steps	10

**Table 3:** Summary of the considered ZOO configuration.

Parameter	Value
Norm ($l_{p}$)	$l_{2}$
Confidence (κ)	0.0
Binary Search Steps	10

**Table 4:** Summary of the considered HopSkipJump configuration.

Parameter	Value
Norm ($l_{p}$)	$l_{\infty }$
Max iterations	40
Binary Search Steps	5

**Table 5:** Summary of the benchmarking datasets considered for this research.

Dataset	Publisher	Year	Attacks	Feature Sets	Size by Records
UNSW-NB15 [23]	Cyber Range Lab of UNSW	2015	10 classes	45	2 million
ToN-IoT [24]	Cyber Range Lab of UNSW	2020	9 classes	31	22 million
Edge-IIoT [25]	IEEE Dataport	2022	14 classes	61	20 million

**Table 6:** Accuracy of selected models among the benchmarking datasets.

Model	UNSW-NB15 Dataset	ToN-IoT Dataset	Edge-IIoT Dataset
SVM	74.90%	97.28%	97.00%
LR	76.57%	97.17%	98.37%
MLP	84.50%	99.97%	99.87%
RF	85.77%	99.98%	99.99%
Voting model	77.78%	97.95%	98.53%
Stacked model	85.48%	99.99%	99.71%

**Table 7:** Adversarial accuracy obtained using the considered models and the UNSW-NB15 dataset [19].

Attacks None		Black-Box (FGSM)	Black-Box (C&W)	0th-Optimization (ZOO)	HopSkipJump (HSJ)
*Model*	Standard Accuracy	Adversarial Accuracy	Adversarial Accuracy	Adversarial Accuracy	Adversarial Accuracy
SVM	74.90%	25.15%	27.22%	74.87%	11.43%
LR	76.57%	39.70%	41.08%	37.14%	09.93%
MLP	84.50%	55.30%	70.10%	40.97%	67.36%
RF	85.77%	64.99%	82.11%	83.60%	83.61%
Voting model	77.78%	28.44%	26.51%	77.69%	11.14%
Stacked model	85.48%	53.98%	66.46%	49.31%	07.81%

**Table 8:** Adversarial accuracy results recorded using the considered models and the ToN-IoT dataset [20].

Attacks None		Black-Box (FGSM)	Black-Box (C&W)	0th-Optimization (ZOO)	HopSkipJump (HSJ)
*Model*	Standard Accuracy	Adversarial Accuracy	Adversarial Accuracy	Adversarial Accuracy	Adversarial Accuracy
SVM	97.28%	39.36%	4.19%	97.25%	1.46%
LR	97.17%	56.41%	83.43%	65.30%	1.18%
MLP	99.97%	59.47%	32.57%	63.75%	6.50%
RF	99.98%	65.61%	69.28%	99.67%	72.14%
Voting model	97.95%	57.32%	2.83%	97.80%	1.51%
Stacked model	99.99%	68.17%	76.87%	75.41%	0.00%

**Table 9:** Adversarial accuracy results obtained for using the selected models on and the Edge-IIoT dataset [21].

Attacks None		Black-Box (FGSM)	Black-Box (C&W)	0th-Optimization (ZOO)	HopSkipJump (HSJ)
*Model*	Standard Accuracy	Adversarial Accuracy	Adversarial Accuracy	Adversarial Accuracy	Adversarial Accuracy
SVM	97.00%	57.29%	50.74%	97.00%	2.39%
LR	98.37%	65.44%	93.09%	70.76%	1.36%
MLP	99.87%	93.20%	84.27%	91.17%	0.22%
RF	99.99%	64.13%	83.64%	99.77%	72.14%
Voting Model	98.53%	68.90%	44.12%	98.50%	1.14%
Stacked Model	99.71%	90.61%	78.40%	94.30%	0.27%

**Table 10:** Summary of the considered feature squeezing configuration.

Parameter	Value
Bit depth	(5)
Clip value	(0,1)

**Table 11:** Summary of the considered adversarial training configuration.

Parameter	Value
Classifier	The assigned target model (i.e., six candidate models)
Attack	The assigned attack for generating AEs (i.e., FGSM. C&W, ZOO, and HopSkipJump)
Number of Epochs	(20)

**Table 12:** Comparison of standard accuracy obtained using the considered datasets.

Dataset	Models	Standard Accuracy	RobEns Standard Accuracy
**UNSW-NB15**	SVM	74%	75%
	LR	76%	76%
	MLP	84%	79%
	RF	85%	86%
	Voting model	77%	77%
	Stacked model	85%	87%
**ToN-IoT**	SVM	97%	97%
	LR	97%	97%
	MLP	99%	99%
	RF	99%	90%
	Voting model	97%	97%
	Stacked model	99%	99%
**Edge-IIoT**	SVM	97%	97%
	LR	98%	98%
	MLP	99%	99%
	RF	99%	100%
	Voting model	98%	98%
	Stacked model	99%	99%

**Table 13:** Performance results obtained using the related works and proposed RobEns framework.

Reference	Datasets	Attack Scenario	Attack Method	Defense Type	Defense Method	Results and Findings
Taheri et al. [5]	CTU-13	White Box	FGSM DeepFool PGD GAN	Single	Adversarial Training	- Robust accuracy less than standard accuracy - No investigation of defense effect over the standard accuracy
Fu et al. [12]	CSE-CIC-IDS2018	White Box	FGSM	Single	Adversarial Training	- Robust accuracy less than standard accuracy - The defense method reduces the standard accuracy significantly
Anthi et al. [13]	Private	White Box	Rule-based Approach	Single	Adversarial Training	- Robust accuracy less than standard accuracy - No investigation of defense effect over the standard accuracy
Ibitoye et al. [14]	BoT-IoT	White Box	FGSM PGD BIM	Single	Feature Normalization	- Robust accuracy is poor compared to standard accuracy - Positive impact on the standard accuracy
Vitorino et al. [15]	IoT-23 Bot-IoT	Black Box	A2PM	Single	Adversarial Training	- Robust accuracy is slight less compared to standard accuracy - Negative impact on the standard accuracy
Proposed RobEns Framework	UNSW-NB15 ToN-IoT Edge-IIoT	Black Box	FGSM C&W ZOO HopSkipJump	Dual	- Feature Squeezing - Adversarial Training	- Framework’s robust accuracy achieve same standard accuracy results - Framework’s standard accuracy is maintained

## Data Availability

All the data have been presented in the main text.
